# Pharmacological and Parenteral Nutrition-Based Interventions in Microvillus Inclusion Disease

**DOI:** 10.3390/jcm10010022

**Published:** 2020-12-23

**Authors:** Changsen Leng, Edmond H. H. M. Rings, Saskia N. de Wildt, Sven C. D. van IJzendoorn

**Affiliations:** 1 Department of Biomedical Sciences of Cells and Systems, Section Molecular Cell Biology, University Medical Center Groningen, University of Groningen, 9713 GZ Groningen, The Netherlands; lengchangsen@hotmail.com; 2 State Key Laboratory of Oncology in South China, Collaborative Innovation Center for Cancer Medicine, Guangdong Esophageal Cancer Institute, Department of Thoracic Surgery, Sun Yat-sen University Cancer Center, Guangzhou 510060, China; 3 Department of Pediatrics, Erasmus University Medical Center, 3015 GD Rotterdam, The Netherlands; e.rings@erasmusmc.nl; 4 Department of Pediatrics, Leiden University Medical Center, 2333 ZA Leiden, The Netherlands; 5 Department of Pharmacology and Toxicology, Radboud Institute Health Sciences, Radboud University Medical Center, 6525 GA Nijmegen, The Netherlands; Saskia.deWildt@radboudumc.nl; 6 Intensive Care and Department of Pediatric Surgery, Erasmus University Medical Center, 3015 GD Rotterdam, The Netherlands

**Keywords:** microvillus inclusion disease, MYO5B, myosin Vb, congenital diarrheal disorder, malabsorption

## Abstract

Microvillus inclusion disease (MVID) is a rare inherited and invariably fatal enteropathy, characterized by severe intractable secretory diarrhea and nutrient malabsorption. No cure exists, and patients typically die during infancy because of treatment-related complications. The need for alternative treatment strategies is evident. Several pharmacological interventions with variable successes have been tried and reported for individual patients as part of their clinical care. Unfortunately, these interventions and their outcomes have remained hidden in case reports and have not been reviewed. Further, recent advances regarding MVID pathogenesis have shed new light on the outcomes of these pharmacological interventions and offer suggestions for future clinical research and trials. Hence, an inventory of reported pharmacological interventions in MVID, their rationales and outcomes, and a discussion of these in the light of current knowledge is opportune. Together with a discussion on MVID-specific pharmacokinetic, -dynamic, and -genetic concerns that pose unique challenges regarding pharmacological strategies, we envision that this paper will aid researchers and clinicians in their efforts to develop pharmacological interventions to combat this devastating disease.

## 1. Introduction

### 1.1. Clinical Presentation

Microvillus inclusion disease (MVID; OMIM #251850) is an autosomal recessive congenital diarrheal disorder [1,2]. From the start of their lives, patients with MVID suffer from unstoppable secretory diarrhea at complete bowel rest (that is, in the absence of enteral feeding). The diarrhea often exceeds that seen in cholera-infected children but is variable between patients. Oral or enteral feeding is not possible and causes massive osmotic diarrhea, resulting in patients’ failure to thrive [1,2].

In order to provide a detailed description of the clinical presentations of MVID, we searched EMBASE and MEDLINE databases using the following search strings: ((microvill* inclusion disease) OR (microvill* atrophy)) AND case report) to collect all published MVID case reports. Seventy valid case reports reporting on 98 patients with MVID were retrieved (Table S1). From these, information on patient gender, gestation, bodyweight at birth, presence or absence of polyhydramnios, day of onset, stool output, fecal analyses, and age at death, among others, were extracted and analyzed (Table 1 and Table 2).

### 1.2. Diagnosis

MVID is diagnosed on the basis of the evaluation of symptoms, the exclusion of more common causes of diarrhea, and the evaluation of intestinal biopsies. Intestinal biopsies of patients with MVID reveal villus hypoplasia in the small intestine without signs of infection or inflammation [1]. Villus enterocytes display intracellular accumulation of periodic acid-Schiff (PAS)-positive material and the brush border enzyme CD10 [3,4]. Electron microscopy reveals microvillus brush border atrophy and the appearance of pathognomonic microvillus inclusions in the cytoplasm of some villus enterocytes [4]. Bi-allelic mutations in the *MYO5B* [5] or *STX3* [6] genes can confirm MVID diagnosis. All features of MVID can also present as part of familial hemophagocytic lymphohistiocytosis, an immune disorder caused by *STXBP2* gene mutations [7].

### 1.3. Pathogenesis

**Figure 1:**
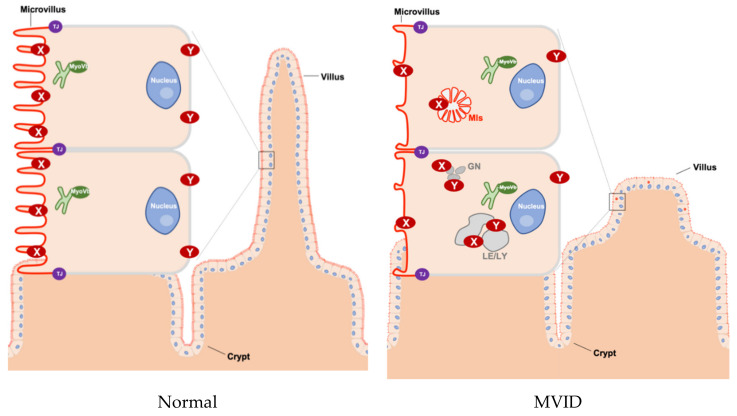
Schematic overview of tissue and cellular characteristics of healthy and MVID intestinal epithelium. In healthy enterocytes, the recycling endosome (green) is located sub-apically and plays an important role in transporting proteins to the plasma membrane (in particular to the apical membrane). However, in MVID that is caused by the loss function of myosin Vb (MyoVb), the villi show hypoplasia and microvilli are atrophic. Moreover, the proteins (marked as X and Y) in the plasma membrane are mislocalized in microvillus inclusions (MIs) or in enlarged late endo/lysosomes (LE/LY) and granules (GN). **X** indicates CD10, DRA, GLUT5, NHE3, SGLT1, sucrase-isomaltase (SI), 5’-nucleotidase (5′NT), alkaline phosphatase (AP), AQP7, CD36, CFTR, DPPIV. **Y** indicates transferrin receptor (Tfr), Na^+^/K^+^ ATPase. TJ: tight junction, MIs: microvillus inclusions, LE: late endosomes, LY: lysosomes, GN: granules.

The proteins that are encoded by the three MVID-associated genes are functionally linked [8,9,10]. *MYO5B, STX3,* or *STXBP2* mutation-driven loss of effective absorptive surface area causes malabsorption and chronic secretory diarrhea [10]. The loss of absorptive surface area includes villus hypoplasia, microvillus atrophy, and the general mislocalization (and in some cases a reduced expression) of brush border proteins. These brush border proteins include those involved in dietary nutrient digestion or absorption (e.g., lactase-phlorizin hydrolase, sucrase-isomaltase, dipeptidyl peptidase IV, glucose/sodium symporter SGLT-1) [11,12] and water resorption (e.g., aquaporins) [11]. Other brush border proteins include those involved in electrolyte transport across the brush border membrane (e.g., the sodium/proton exchange protein SLC9A3 (NHE3) and the bicarbonate/chloride exchange protein SLC26A3 (DRA). The loss of *MYO5B, STX3*, or *STXBP2* in intestinal cells, intestinal organoid cultures, or mice has been causally related to the mislocalization of these brush border proteins [6,9,11,13,14] and to the loss of microvilli [15,16]. The consequences for the localization of the chloride/bicarbonate transporter (CFTR) are less clear. CFTR maintains its expression and brush border localization in the enterocytes of m*yo5b*-depleted mice and some—but not all—patients [8,14,17]. All cellular defects appear less pronounced in the crypt area when compared to the villus area [18]. This has led to the suggestion that a defect in intestinal epithelial cell differentiation—and a resultant immature epithelium composed of predominantly secretory crypts and few absorptive villi that show (residual) chloride secreting capacity but cannot absorb sodium and chloride—underlies the symptoms. For more details on MVID pathogenesis, the reader is referred to the recent excellent review articles [19,20]. A cartoon illustrating key tissue and cellular defects in MVID is presented in Figure 1.

### 1.4. Relation to Other Congenital Diarrheal Disorders

MVID displays characteristics of other diarrheal disorders. For example, the proteins DRA and NHE3—which show reduced expression and/or are mislocalized in MVID enterocytes [11,17,21]—are mutated in congenital chloride diarrhea (CCD) and congenital sodium diarrhea (CSD), respectively. From all collected case reports (Table S1), we retrieved those that mentioned at least one data value regarding stool output, electrolytes, osmolarity, or pH value (Table 1). Inspection of fecal electrolyte compositions from the retrieved MVID case reports (Table 2) indicate that the average patient with MVID (fecal Na^+^ 89 mEq/L, Cl^−^ 75 mEq/L) does not typically fulfil the criteria for the diagnosis of either CSD (fecal Na^+^ > 100 mEq/L, Cl^−^ > 40 mEq/L) or CCD (fecal Na^+^ > 60 mEq/L, Cl^−^ > 120 mEq/L). Further, unlike in CSD and CDD, polyhydramnios (in utero diarrhea) is not common in MVID. However, we found it to be reported in 7 out of 29 cases (24%; Table 1) for which the presence or absence of polyhydramnios was mentioned. Upon oral or enteral feeding, MVID resembles congenital osmotic diarrhea (characterized by high fecal sodium losses, e.g., congenital glucose-galactose intolerance and congenital sucrase-isomaltase deficiency), presumably due to the reduced brush border localization of SGLT-1 and sucrase-isomaltase (SI). MVID thus presents as a mix of other congenital diarrheal and malabsorption disorders.

### 1.5. Current Treatment of MVID

Treatment options are limited. First treatment addresses immediate life-threatening dehydration and metabolic acidosis. Oral rehydration solution (glucose-mediated sodium absorption) is ineffective because of the absence of brush border SGLT-1. Patients with MVID typically require life-long total parenteral nutrition (TPN), also known as hyperalimentation, to compensate for fecal fluid and salt losses and provide nutrients to support growth [22]. Intestinal transplantation is an option [23], but only for specific cases because of its lower 5-year survival rate (≈60%).

## 2. Parenteral Nutrition-Based Interventions in MVID

While life-saving at first, TPN does not stop the diarrhea and most patients die during infancy because of TPN-associated complications [24]. TPN-related complications are the predominant cause of death in MVID. The most frequent TPN-related complication that causes death of patients with MVID is catheter-related sepsis. Another life-threatening complication of long-term TPN in patients is liver failure (steatosis, cholestasis progressing to cirrhosis) [23,25]. This has been attributed to hepatotoxic effects of soybean lipids (rich in omea-6 fatty acids) in the TPN. In a murine model, intravenous administration of omega-3 fatty acid-enriched fish oil-based lipids resulted in less steatosis when compared to omega-6 fatty acid-rich soybean oil-based lipids. Interestingly, for five patients with MVID, a reduction of liver symptoms was reported when soybean oil-based lipids in the TPN were replaced by fish oil-based lipids [26,27]. While further research is clearly needed to determine which formula will reduce complications, TPN will not be a part of the cure of MVID.

## 3. Pharmacological Interventions in MVID

While no clinical trials for MVID have been reported in the literature or databases (www.clinicaltrials.gov), several pharmacological treatments have been tried with individual patients with MVID and reported in published case reports. In order to review these, we manually screened each of the collected case studies (Table S1) for reports of non-routine pharmacological intervention. This yielded 15 articles reporting a total of 35 patient treatments involving in total 8 different pharmacological interventions (Table 3). The reported pharmacological interventions could be divided from a conceptual point of view into three categories: (i) drugs that stimulate enterocyte proliferation and/or differentiation, (ii) anti-diarrheal drugs that modulate ion balance across the enterocytes’ brush border membrane, and (iii) other anti-diarrheal drugs. For all reported treatments, their rationales and outcomes are discussed below.

### 3.1. Drugs That Stimulate the Proliferation and/or Differentiation of Enterocytes

#### 3.1.1. Epidermal Growth Factor (EGF)

EGF is a naturally secreted peptide that binds to the EGF receptor on the basal surface of the enterocytes. Binding to the receptor results in the activation of signaling pathways that stimulate cell proliferation and maturation. Given the severe villus hypoplasia in the small intestine of patients with MVID, the rationale for use in MVID was that EGF may stimulate the proliferation of cells in the crypt and in this way would yield more enterocytes to repopulate and thereby regenerate absorptive villi.

Drumm et al [28] reported two patients with MVID that received continuous intravenous infusion at 100 ng/kg/h. The patients then received the same dosage of EGF for 5 days (case 1) and for 21 days (case 2), followed by the same dose for 21 days intravenously (case 1) or by continuous enteral infusion (case 2). Outcome measures were 24 h stool collections, disaccharidase activity in jejunal biopsy homogenates, and mucosal epithelial morphometry. The observed increase in crypt cell proliferation indicated that EGF displayed functional activity. However, EGF did not result in increased villus length or clinical improvement. Walker-Schmitz et al [29] reported that one patient received a dosage of 100 ng/kg/h EGF for 2 days with 5 days rest period between two courses. Outcome measures were stool volume, small-bowel mucosal morphometry, and epithelial cell kinetics. However, only an increase in crypt cell proliferation was found without other improvements. In a later study, Beck et al [30] tried EGF (100 ng/kg/h for 2 weeks) with one MVID patient and reported some microvillus restoration but no reduction is stool volume.

#### 3.1.2. Steroids

Steroids, namely, (gluco)corticosteroids such as prednisolone or hydrocortisone, have been reported to have beneficial effects on the maturation and function of the small intestine in animal models [31]. Six studies reported treatment of a total of eight patients with MVID with steroids but reported no beneficial effects on stool volume [28,30,32,33,34,35].

### 3.2. Anti-Diarrheal Drugs That Modulate Electrolyte Transport across the Brush Border Membrane

#### 3.2.1. Somatostatin/Octreotide

Somatostatin is a naturally occurring hormone. Somatostatin binds to somatostatin receptor, which is a G_i_-protein-coupled transmembrane receptor. When activated, these inhibit adenylate cyclase (AC) [36]. AC is an enzyme that, downstream of G_α_-coupled receptors at the cell surface, produces adenosine 3’,5’-cyclic monophosphate (cAMP). cAMP, in turn, stimulates the activity and brush border abundance of the CFTR protein and inhibits the function of the NHE3 and DRA proteins [37]. Inhibition of adenylate cyclase, therefore, may be expected to reduce CFTR-mediated secretion of chloride and stimulate NHE3-mediated sodium transport across the brush border surface. Because somatostatin is rapidly degraded by peptidase enzymes in cells and plasma and is poorly absorbed in the gut, continuous intravenous infusion is required but impractical for long-term management. Therefore, the hydrophilic octapeptide octreotide, which is a long-acting somatostatin analogue, has also been used for stool output reduction in patients with high-output secretory diarrhea of various causes [38].

Couper et al [39] reported in one MVID patient a 40% stool volume decline and reductions in the output of stool electrolytes following subcutaneous octreotide injections (100 μg two times a day for 14 days). In contrast, Schofield et al [40] reported in one MVID patient no beneficial effect of somatostatin. Five other studies reported octreotide as intervention with eight patients with MVID. Beck et al [30] and Raafat et al [33] found no effect of octreotide on stool volume in two patients with MVID per study. Three other studies mentioned octreotide been tried with one patient with MVID per study and reported no beneficial effects [41,42,43]. Unfortunately, in these latter studies the study protocol and outcome measures were not specified. No follow-ups of any of these treated patients have been reported.

#### 3.2.2. Racecadotril

Racecadotril (acetorphan) is a lipophilic thiorphan derivative and rapidly converted to thiorphan, a potent inhibitor of enkephalinase [44]. Inhibition of enkephalinase prevents the degradation—and thereby increases the biological half-life—of enkephalins, which are endogenous opioid peptides secreted by myenteric and submucosal neurons in the digestive tract. These enkephalins bind and activate δ-opioid receptor on the basal surface of enterocytes, thereby inhibiting AC activity and reducing cAMP levels in the intestinal epithelium. Similarly to somatostatin and octreotide, a resultant reduction in cAMP levels was expected to inhibit CFTR-mediated chloride output and stimulate sodium absorption and, thereby, reduce fluid secretion. Tran et al [45] treated one patient with MVID with racecadotril (1.5 mg/kg; three times a day) and used stool frequency and the Bristol stool chart indications as outcome measures. Mean daily number of stools was reduced and stool consistency improved. Cessation of nocturnal stooling and improved sleep and appetite was reported by the patient’s parents. Withdrawal resulted in more frequent watery stools, which was once again improved after reintroduction of the drug. While promising, since this report, there have been no additional studies reporting on racecadotril in MVID.

#### 3.2.3. Loperamide

Loperamide is an opioid receptor agonist and acts on the μ-opioid receptors in the myenteric plexus of the large intestine, thereby decreasing the activity of the myenteric plexus and consequently intestinal motility [46,47]. This results in reduced stool transit time in healthy people, allowing more time for water homeostasis and stool thickening. Loperamide can be prescribed for adults and children above 2 years of age for treatment of acute non-specified diarrhea. The currently prescribed dose of oral loperamide solutions for the treatment of diarrhea in children of 2–5 years of age (13–20 kg) is a maximal 3 mg/day. Four studies mentioned loperamide intervention with five patients with MVID at varying doses (0.1–1.0 mg/kg/day). One patient (receiving the lowest reported dose of 0.1 mg/kg/day) was reported to display a—non-specified—significant reduction in stool volume [34], whereas no effect was observed in the other patients [32,33,34,45].

### 3.3. Other Anti-Diarrheal Drugs

#### Cholestyramine

Cholestyramine is a bile acid sequestrant. It is used in the clinic for the symptomatic control of bile acid-induced diarrhea due to short bowel syndrome [48]. One study reported a threefold reduction (from 150 to 50 mg/kg/day) in stool volume upon treatment of a patient with MVID with an unspecified dose of cholestyramine [30]. Two other studies (also with unspecified doses) with each one patient with MVID found no beneficial effect [32,42].

## 4. Discussion and Future Perspectives

### 4.1. Rational Approaches for Pharmacological Treatment of Patients with MVID

#### 4.1.1. The AC-Inhibiting Drugs Somatostatin/Octreotide, Loperamide and Racecadotril

Variable, sometimes promising but overall unsatisfying treatment response with regard to reducing stool volume has been observed in patients with MVID treated with AC-inhibiting compounds (Table 3). Recent analyses of ion transporter deficits in tissues of patients with MVID, MVID cell lines, and animal models [11,17,21,49], in conjunction with new insights in the cAMP-mediated regulation of these transporters [50,51], may offer mechanistic explanations for the variable and overall limited effectiveness of these compounds.

##### Efficacy of AC-Inhibiting Drugs in MVID

AC-inhibiting drugs have been shown to be effective in situations where a (pathogen-induced) increase in the concentration of cAMP causes secretory diarrhea. AC-inhibiting drugs can (i) inactivate or stimulate the endocytic removal of NHE3 from the brush border surface and thereby inhibit sodium absorption [52], and (ii) stimulate CFTR activity and chloride secretion. In MVID enterocytes, the majority of NHE3 is mislocalized to cytoplasmic vesicles, and *MYO5B* mutations impair NHE3 trafficking to the brush border surface. This likely limits the expected inhibitory effect of AC-inhibiting compounds on NHE3 and stimulatory effect on sodium absorption [52]. Effects of AC-inhibiting compounds on DRA are hampered by the severely reduced expression of the DRA protein in MVID enterocytes [17]. Loss of DRA activity and elevated luminal chloride will also further reduce NHE3 function, as is seen in CCD patients carrying DRA mutations. Conversely, in the absence of NHE3, DRA function becomes more dependent on CFTR [53], the remaining target of AC-inhibiting drugs. CFTR has been reported to maintain its brush border localization (and even be hyperactivated [54]) in some patients with MVID [17], but not in other patients [9,55]. AC-inhibiting compounds may therefore show limited effect in patients with MVID of which enterocytes show mislocalization of CFTR to the cytoplasm. AC-inhibiting compounds could be effective in patients with MVID with (residual) CFTR in the brush borders of their enterocytes. The efficacy will likely depend on the extend of the expression and activity of brush border CFTR. Postnatal surges of glucocorticoids have been suggested to hyperactivate brush border CFTR in MVID enterocytes [54], raising the possibility that AC-inhibiting compounds may be more effective in this period.

##### Adverse Effects and Limitations of AC-Inhibiting Drugs in MVID

Although no adverse effects of any of the reported drugs were reported in the case studies, the use of octreotide at high doses or for long-term use in pediatric patients is not without risk. Indeed, one study reported serious adverse effects in 19% (4 out of 21) of cases [32]. Loperamide is currently not prescribed for children under the age of 2 years due to the risks of respiratory depression and serious cardiac adverse reactions.

##### Novel Anti-Diarrheal Drugs for MVID

Novel anti-diarrheal drugs targeted against specific brush border transporter proteins are being developed [56,57], but these have thus far not been tried in MVID. For MVID, the efficacy of CFTR-targeting drugs will depend on the amount and activity of CFTR present at the brush border surface, which varies between patients [9,17,55]. Similarly, the effectivity of NHE3-activating peptides depends on the amount of NHE3 at the cell surface, which is limited in most MVID cases where this protein was investigated. The effectivity of drugs or probiotics—such as *Lactobacillus acidophilus*—aimed at stimulating NHE3 or DRA gene expression [58,59], is therefore likely to be limited by the inability of newly synthesized NHE3 and DRA proteins to maintain sufficient localization at the brush border due to the *MYO5B* mutations [14,17,59]. Interestingly however, recent studies in mice suggested the existence of myosin Vb-independent trafficking routes for brush border proteins including NHE3, which can be stimulated by treatment of enterocytes with lysophosphatidic acid [60,61,62]. Thus, a drug-stimulated increase in total amount of these transporters at the brush border may be a promising treatment option.

##### A Genotype–Phenotype Relationship in Treatment Response?

We found that significant variations have been reported in stool output (50–300 mL/kg/day) and/or in fecal electrolyte compositions between patients (Table 1 and Table 2). Whether there is a relationship between the severity of diarrhea and treatment response is not known. The cause of interpatient variations in stool output, stool electrolyte composition, and the precise relative expression and localization pattern of the different ion transporters at the brush border is also not known. Possibly, these reflect the patients’ *MYO5B* mutation. More than 60 unique *MYO5B* mutations have been identified, with each family carrying distinct sets of *MYO5B* mutations (www.mvid-central.org) [10,15]. These can differently affect the encoded myosin Vb protein and, conceivably, brush border protein localization and drug response. A genotype-dependency for the therapeutic efficacy of butyrate was recently reported in CCD patients [63]. Future genotype–phenotype correlation studies may shed light on and help predicting treatment responses. There is much interest in the use of patient-specific intestinal organoids for studying genotype–phenotype correlations [64]. While promising, care should be taken as cultured patient organoids may not accurately reflect rapidly changing brush border transporter (e.g., NHE3) profiles during early childhood development.

#### 4.1.2. The Enterocyte Proliferation- and Differentiation-Stimulating Drugs EGF and Steroids

It should be emphasized that MVID is both a diarrheal and malabsorption disorder. The prevention of diarrhea in MVID, as such, is likely to improve the management of electrolyte supplementation and reduce the risk of metabolic decompensation. However, it is as such not expected to reduce patients’ dependency on TPN and therewith associated mortality. Indeed, chronic diarrhea or malabsorption and villus hypoplasia are not typically correlated, as evidenced by the normal intestinal architecture in CSD, CCD, and congenital glucose-galactose malabsorption patients. Resolving only diarrhea may therefore not be expected to normalize intestinal architecture (i.e., restore villus length).

The remaining villus hypoplasia thus precludes an optimal absorptive epithelium to accommodate oral or enteral nutrient intake. Therefore, in conjunction with the search for effective antidiarrheal treatment, efforts should be directed at restoring villus length. Thus far, drugs such as EGF could stimulate enterocyte proliferation and some differentiation but proved ineffective to ameliorate clinical symptoms in all patients with MVID studied. It was proposed that the villus hypoplasia in MVID reflects (in part) a degeneration triggered by a hitherto unknown factor [28]. A recent study in germline *Myo5b* knockout mice, which showed normal intestinal villi length before birth and villus hypoplasia within days after birth [65], suggests a postnatal factor. The further identification of these postnatal factors may unlock new territories for pharmacological interventions.

### 4.2. Practical Aspects of Pharmacological Treatment of Patients with MVID

Thus far, most drugs have been provided via intravenous or subcutaneous injections. This is not the most practical for children with MVID as they likely require life-long treatment. Orally administered drugs would be more practical. However, oral bioavailability of pharmacological drugs, in particular hydrophilic drugs that require active trans-mucosal transport, is a critical parameter especially in MVID. Indeed, *MYO5B* mutations cause the downregulation and/or mislocalization of a wide variety of brush border transporters. It should therefore be anticipated that those brush border transporters required for the uptake of hydrophilic drugs such as SLC15A1 (PepT1) may show reduced brush border membrane expression or, in case of proton-dependent transporters, show reduced activity due to impaired NHE3- or v-ATPase-mediated proton secretion in MVID enterocytes. Furthermore, cell surface expression of drug efflux transporters such as the multidrug resistance protein ABCC2 (MRP2) has been shown to be inhibited by *MYO5B* mutations in the liver of patients with MVID [66,67]. While these drug-transporting cell surface proteins thus far have not been studied in the MVID intestine, alterations in these can be expected to have consequences for pharmacokinetics and optimal drug dosage. Convectional washout of drugs because of the continuous severe diarrhea poses an additional pharmacokinetic hurdle for the oral and enteral administration of both hydrophilic and lipophilic drugs.

With regard to treatment duration and timing, it is recommended that treatment duration is at least 5 days because of the relatively high turnover time of enterocyte. An unsuccessful intervention in a MVID patient with mesenchymal stem cells has been attributed to a treatment that is too short [68]. The timing of pharmacological interventions aimed at specific brush border transporters in young children is important as such transporters show dynamic changes in expression during postnatal development, thereby affecting drug efficacy. In this regard, therapeutic targets identified on the basis of results of patient biopsy analyses that were taken prior to the diagnosis may no longer reflect the situation in the patient’s intestine at (later) moments of drug intervention.

### 4.3. Suggestions for Future Reporting

MVID is a rare disease and large randomized controlled trials are not foreseen. Crossover placebo-controlled n-of-1 trials may be an appropriate choice given the limited number of available patients with MVID and the chronicity of the condition, provided that the expected treatment response is stable, the onset of the treatment effect is quick, and that carryover effects (depending on drug elimination half-life) are controlled.

Likely, reports of pharmacological interventions will thus continue to typically involve one or few patients per published report. Treatment recommendations will be largely based on clinical experience and anecdotal reporting in the medical literature. To facilitate future meta-analyses of these single patient studies, we recommend that publications include more systematic descriptions of the study protocols (e.g., dosage, dose rationale, intervention duration, administration route, outcome measures, including efficacy and safety parameters) and clinical details (e.g., early/late onset MVID, age, mutation details, baseline measures (e.g., volume, frequency and consistency of diarrhea, fecal electrolytes)).

## Figures and Tables

**Table 1:** Clinical details from published microvillus inclusion disease (MVID) case reports.

PMID#	Gender	Gestation (Week)	Birth Body Weight (g)	Poly Hydramnios	Onset (Day)	Stool Output (ml/kg/d) *	Fecal Electrolyte (mmol/L)			Fecal Osmolarity (mOsm/kg *)	Fecal PH Value *	Dead/Alive	Follow-up
							Na *	Cl *	K *				
28842815	Male	at term	3500		3		78	64	7.3		7	Dead	1 month
	Female	34	2000		5						7	Dead	36 days
29546954	Male	36		No	10	190	120	67		30		Alive	36 months
25111220	Female				150		15					Alive	132 months
23525737	Male	at term			6	150						Alive	168 months
23354788	Female	35	2330	Yes	1		85	78	22			Dead	7 months
23226823	Female	at term		No	3	100						Dead	4 months
22318102	Female	35	2320	Yes	3		35	21				Dead	23 days
22197941	Female	at term	2900	Yes	1	100	78	42	40	11		Alive	4 days
22152886	Male	36			4	148						Alive	41 months
21968248	Male	at term	2734	No	3	175	84	68	13			Alive	3 months
21299349	Male	35			3						6	Dead	2 months
18277898	Male	34	2450	No	8		112	113	21.6	292		Alive	12 days
17418172	Male	31			3	100	139	105	4.7	279		Alive	12 days
15456973	Female	at term	2530		1	200						Dead	1.5 months
11783915	Male	at term			6	115	95	95	30	270	9	Alive	5 months
11414303	Male	36	2700	NO	2	200	100	60	17.5			Alive	4 months
11251929	Male	35	3720	Yes		300						Alive	3 days
11173328	Male	36	2740	No	3		76	79	39			Dead	6 months
10941974	Female	at term	3510		11		108	55	11.9	330		Alive	39 months
10941971	Female	34	2100	No	6	175						Alive	24 months
9932857	Female	36			10	170				261		Alive	9 months
9880458	Male	at term	3500		6	95	110	85	7			Alive	24 months
	Male	at term	2600		6	135	81	44	2			Alive	96 months
	Male	36	3300		6	150	115	96	5			Alive	84 months
	Female	at term	3160		4	175	107	84	18			Alive	48 months
	Female	at term	2700		4	100	6		27			Dead	36 months
9844114	Male	at term	3300	No	1	75						Alive	132 months
9822319	Male	36	3090	No	1	100	119	111	14		6.5	Alive	3 months
	Male				14	150	105	74	12	281		Alive	9 months
9740207	Male	at term	3900	No	14	178	105	74	12.1	281	8	Dead	18 months
9364305	Female	36	2700	No		175						Alive	3 months
9323563	Male	33	2950	Yes	1		99		12	240		Dead	7 months
8732907	Male	at term	3350	No	7	50						Alive	7 months
7959671	Female	at term	4100	No	14	60	91					Dead	39 months
	Male	at term	4200	No	14	50	100					Dead	5 months
	Male	at term	3800	No	7		95					Alive	58 months
8067796	Male	at term	3325	No	1	166	58	36	15	309		Dead	9 days
	Male	35	2880	No	2	200						Dead	4 months
8032396	Female	35	2810	No	3	120	104		19	240		Alive	7 months
1319670	Female	at term	2700	No	4	200	6		27			Dead	37 months
1660676	Male	at term	3530	Yes	1	150	103	89	19			Alive	72 months
	Male	at term	3300	Yes	1	150	122	102	19.4			Alive	9 months
2759484	Female	at term	2300	No	3	85	100	82	29			Alive	13 months
3977385	Female	at term	2500	No	2		91					Dead	6 months
	Female	34	2200	No	4		93					Dead	6 months
25635218	Female	36	2800		1	120	83				8	Dead	9 months
	Female	36			7	100						Alive	13 months

This table includes patients from the collected case reports (Table S1) that have at least one data value reported on stool output, fecal electrolyte or osmolarity, or pH value. Forty-eight patients, including 27 males and 21 females (the ratio male/female was 1.29), are presented. Among these, 24 patients were born at term, 32 patients were born later than 39 weeks of pregnancy, and 2 patients were not reported. Seven patients were reported with polyhydramnios during pregnancy; pregnancy for 22 patients was not associated with polyhydramnios, and for 19 patients, the presence of absence of polyhydramnios was not reported. The median onset of diarrhea was at 4 days. For 37 (out of 46) patients (80.4%), the onset of diarrhea was in the first week. Nineteen patients were reported to have died and 29 patients were still alive at the moment of reporting. The median age of death was 6 months. * Reference values: fecal sodium: 10-30 mmol/L; stool output: 0–10 mL/kg/day, 30 mmol/L; fecal potassium: 75 mmol/L; fecal chloride: 10–30 mmol/L; fecal osmolality: 290 mOsm/kg; fecal pH: 7–8.

**Table 2:** Summary of the clinical details from published MVID case reports.

	Birth Body Weight (g) *n* = 37	Stool Output (ml/kg/d) *n* = 35	Fecal Electrolyte (mmol/L)			Fecal Osmolarity (mOsm/kg) *n* = 12	Fecal PH Value *n* = 7
			Na^+^ *n* = 34	Cl^-^ *n* = 23	K^+^ *n* = 25		
Minimum	2000	50	6	21	2	11	6
Maximum	4200	300	139	113	40	330	9
Average	2987	140.2	88.8	75.0	17.8	235.3	7.36

The minimum, maximum, and average values of birth body weight, stool output, fecal features from Table 1 are displayed.

**Table 3:** A list of patients who were treated with pharmacological interventions (retrieved from Table S1). This yielded 15 unique articles reporting a total of 35 patient treatments involving 8 different pharmacological interventions. IV: intravenous injection; SC: subcutaneous injection; Bid: two times per day; Tid: three times per day; Qid: four times per day; n.r.: not reported.

Drug Name	Protocol	Outcome Measures	Result	Patients Number	PMID
EGF	100 ng/kg/h for two 6-day with a 5-day rest period between two courses	Stool volume, small-bowel mucosal morphometry and epithelial cell kinetics	No effect except mitotic index in duodenal crypt increased	1	2866310
EGF	100 ng/kg/h (IV) for 5 days, then followed by same dose for 21 days intravenously	24 h stool collections, disaccharidase activity in jejunal biopsy homogenates and mucosal epithelial morphometry	No effect except mitotic index in duodenal crypt increased	1	2891946
EGF	100 ng/kg/h (IV) for 21 days, then followed by same dose for 21 days continuous enteral infusion	24 h stool collections, disaccharidase activity in jejunal biopsy homogenates and mucosal epithelial morphometry	No effect except mitotic index in duodenal crypt increased	1	2891946
EGF	100 ng/kg/h (IV) for 2 weeks	Stool volume and small-bowel mucosal morphometry	No effect except population of microvilli increased	1	9364305
Somatostatin	100 μg (SC) Bid for 21 days	Stool volume	Decreased from 210 mL/kg/day to 150 mL/kg/day	1	2759484
Somatostatin	n.r.	Stool volume	No effect	1	1660676
Somatostatin	n.r.	Stool volume	Mild decreased	1	1319670
Somatostatin	n.r.	n.r	No effect	1	8114773
Somatostatin	n.r.	n.r.	No effect	1	9323563
Somatostatin	n.r.	n.r.	No effect	1	9880458
Octreotide	100 μg (SC) Bid for 14 days	Stool volume	Decreased from 275 mL/kg/day to 161 mL/kg/day	1	2759484
Octreotide	n.r.	n.r.	No effect	1	1993505
Octreotide	n.r.	n.r.	No effect	2	7959671
Octreotide	4 μg/kg/day	Stool volume	No effect	2	9364305
Octreotide	n.r.	n.r.	No effect	1	11800313
Octreotide	n.r.	n.r.	No effect	1	25635218
Loperamide	1 mg/kg/day	n.r.	No effect	1	3977385
Loperamide	0.1 mg/kg/day	Stool volume	Decreased remarkably	1	3977385
Loperamide	n.r.	Stool volume	No effect	1	1660676
Loperamide	n.r.	n.r.	No effect	1	7959671
Loperamide	0.2 mg/kg Qid	Stool frequency, Bristol stool chart	No effect	1	27682357
Steroid	n.r.	Stool volume	No effect	1	1660676
Steroid	n.r.	n.r.	No effect	1	7959671
Steroid	2 mg/kg/day for 3 weeks	Stool volume	No effect	1	9364305
Prednisolone	n.r.	n.r.	No effect	1	3977385
Dexamethasone	Oral	Stool volume	No effect	1	3977385
Adrenocorticotrophic hormone	n.r.	n.r.	No effect	1	3977385
Hydrocortisone	IV for 4-week	Stool volume	No effect	1	2891946
Glucocorticosteroids	n.r.	n.r	No effect	1	8114773
Cholestyramine	n.r.	Stool volume	No effect	1	1660676
Cholestyramine	n.r.	Stool volume	Decreased from 150 mg/kg/day to 50 mg/kg/day	1	9364305
Cholestyramine	n.r.	n.r.	No effect	1	11800313
Pentagastrin	n.r.	n.r	No effect	1	8114773
Racecadotril	1.5 mg/kg Tid	Stool frequency, Bristol stool chart	The mean daily number of stools fell from 6.5 to 2.1 and stool consistency improved to Bristol type 6.	1	27682357
Mesenchymal stem cells	1*10^6^ U transduodenal and 2*10^6^ U (IV)	Fluid and electrolyte requirements	No effect except blood stream infections were reduced	1	

## Supplementary Materials

The following are available online at https://www.mdpi.com/2077-0383/10/1/22/s1, Table S1: Clinical details from published MVID case reports.
