# Supplementary material for: Pharmacological and Parenteral Nutrition-Based Interventions in Microvillus Inclusion Disease
Source: J Clin Med. 2020 Dec 23;10(1):22. doi: 10.3390/jcm10010022 (PMC7794843; doi:10.3390/jcm10010022)
Supplement: Supplementary file 1 [file jcm-10-00022-s001.pdf]

Supplementary table 1. Clinical details from published MVID case reports.

References	PMID#	Patients number	Gender	Gestation (week)	Birth body weight (g)	Poly hydra mnios	Onset (day)	Stool output (ml/kg/d)	Feces electrolyte (mmol/L)			Feces osmolarity (mOsm/kg)	Feces PH value	Dead/ Alive	Follow-up
									Na^+	Cl^-	K^+				
Lingaldinna et al. (2017)	28842815	1	Male	38	3500		3		78	64	7.3		7	Dead	1 month
		2	Female	34	2000		5						7	Dead	36 days
Schoen et al. (2017)	29546954	1	Male	36		No	10	190	120	67		30		Alive	36 months
Perry et al. (2014)	25111220	1	Female				150		15					Alive	132 months
Burgis et al. (2013)	23525737	1	Male	at term			6	150						Alive	168 months
Siahanidou et al. (2013)	23354788	1	Female	35	2330	Yes	1		85	78	22			Dead	7 months
AI-Sinani et al. (2012)	23226823	1	Female	at term		No	3	100						Dead	4 months
Thomas et al. (2012)	22318102	1	Female	35	2320	Yes	3		35	21				Dead	23 days
Vora et al. (2012)	22197941	1	Female	37	2900	Yes	1	100	78	42	40	11		Alive	4 days
Fuchs et al. (2011)	22152886	1	Male	36			4	148						Alive	41 months
Shahid et al. (2012)	21968248	1	Male	at term	2734	No	3	175	84	68	13			Alive	3 months
Khubchandani et al. (2011)	21299349	1	Male	35			3						6	Dead	2 months
Gathungu et al. (2008)	18277898	1	Male	34	2450	No	8		112	113	21.6	292		Alive	12 days
Amosu et al. (2007)	17418172	1	Male	31			3	100	139	105	4.7	279		Alive	12 days
Kucinskiene et al. (2004)	15456973	1	Female	37	2530		1	200						Dead	1.5 months
Mierau et al. (2001)	11783915	1	Male	at term			6	115	95	95	30	270	9	Alive	5 months
Ruemmele et al. (2001)	11414303	1	Male	36	2700	NO	2	200	100	60	17.5			Alive	4 months

Kennea et al. (2001)	11251929	1	Male	35	3720	Yes		300						Alive	3 days
Wilson et al. (2001)	11173328	1	Male	36	2740	No	3		76	79	39			Dead	6 months
Croft et al. (2000)	10941974	1	Female	at term	3510		11		108	55	11.9	330		Alive	39 months
Bunn et al. (2000)	10941971	1	Female	34	2100	No	6	175						Alive	24 months
Heinz-Erian et al. (1999)	9932857	1	Female	36			10	170				261		Alive	9 months
Pohl et al. (1999)	9880458	1	Male	39	3500		6	95	110	85	7			Alive	24 months
		2	Male	38	2600		6	135	81	44	2			Alive	96 months
		3	Male	36	3300		6	150	115	96	5			Alive	84 months
		4	Female	39	3160		4	175	107	84	18			Alive	48 months
		5	Female	37	2700		4	100	6		27			Dead	36 months
Kagitani et al. (1998)	9844114	1	Male	38	3300	No	1	75						Alive	132 months
Michail et al. (1998)	9822319	1	Male	36	3090	No	1	100	119	111	14		6.5	Alive	3 months
		2	Male				14	150	105	74	12	281		Alive	9 months
Randak et al. (1998)	9740207	1	Male	at term	3900	No	14	178	105	74	12.1	281	8	Dead	18 months
Beck et al. (1997)	9364305	1	Female	36	2700	No		175						Alive	3 months
Assmann et al. (1997)	9323563	1	Male	33	2950	Yes	1		99		12	240		Dead	7 months
Herzog et al. (1996)	8732907	1	Male	at term	3350	No	7	50						Alive	7 months
Raafat et al. (1994)	7959671	1	Female	at term	4100	No	14	60	91					Dead	39 months
		2	Male	at term	4200	No	14	50	100					Dead	5 months
		3	Male	at term	3800	No	7		95					Alive	58 months
Nathavitharana et al. (1994)	8067796	1	Male	38	3325	No	1	166	58	36	15	309		Dead	9 days
		2	Male	35	2880	No	2	200						Dead	4 months
Nizet et al. (1994)	8032396	1	Female	35	2810	No	3	120	104		19	240		Alive	7 months

Schofield et al. (1992)	1319670	1	Female	37	2700	No	4	200	6		27			Dead	37 months
Bell et al. (1991)	1660676	1	Male	at term	3530	Yes	1	150	103	89	19			Alive	72 months
		2	Male	37	3300	Yes	1	150	122	102	19.4			Alive	9 months
Couper et al. (1989)	2759484	1	Female	at term	2300	No	3	85	100	82	29			Alive	13 months
Phillips et al. (1985)	3977385	1	Female	37	2500	No	2		91					Dead	6 months
		2	Female	34	2200	No	4		93					Dead	6 months
Mendes et al. (2014)	25635218	1	Female	36	2800		1	120	83				8	Dead	9 months
Elena et al.		1	Female	36			7	100						Alive	13 months
Sadiq et al. (2019)	31559144	1	Female	35	2445	No	1							Alive	1 month
Comegna et al. (2018)	30564347	1	Male	36	2820		1							Dead	23 months
		2	Male	37	3280	Yes	1							Dead	7 months
Khalsi et al. (2018)	30364420	1	Male	35	3030	No	5							Dead	3 months
Alsaleem et al. (2017)	29282386	1	Male	at term		No	3							Alive	1 month
Bulut et al. (2017)	28707991	1	Male	at term	3300	No	2							Alive	5 months
Tran et al. (2017)	27682357	1	Male											Alive	48 months
Perry et al. (2014)	25111220	1	Female	36	2950	No	2							Alive	36 months
		2	Male	at term	3290		7							Alive	24 months
		3	Male	at term	3570	Yes	42							Alive	12 months
		4	Male	at term	3360	No	5							Alive	144 months
		5	Male	40	2350	No								Alive	156 months
		6	Male	31	1645		420							Alive	288 months
		7	Male				150							Alive	336 months
Wiegerinck et al. (2014)	24726755	1	Female				2							Alive	12 months

		2	Male				14	Alive	18 months
Oatman et al. (2014)	23648791	1	Male				30	Alive	132 months
Chiang et al. (2015)	23608388	1	Male	36			1	Dead	5 months
Golachowska et al. (2012)	22441677	1	Male	at term			3	Alive	60 months
		2	Male				60	Alive	60 months
Fuchs et al. (2011)	22152886	1	Male	37			1	Alive	48 months
		2	Male	37			3	Alive	48 months
Chen et al. (2011)	22030065	1	Female	23	634	No		Dead	6 months
Chen et al. (2010)	21199752	1	Male	36	3355	Yes	1	Dead	6 months
Rund et al. (2006)	16454574	1	Male	35			1	Alive	1.5 months
Weeks et al. (2003)	14708724	1	Female					Alive	8 months
Gambarara et al. (2003)	14697977	1	Male				2	Alive	165 months
		2	Male					Alive	36 months
		3	Female				2	Alive	132 months
		4	Male				1	Alive	13 months
Martinez et al. (2002)	12028658	1	Female	35	2110		1	Alive	4 months
Goldman et al. (2002)	11903944	1	Male				30	Alive	36 months
Levental et al. (2002)	11883547	1	Female				21	Alive	60 months
Ameen et al. (2000)	11208062	1	Female	at term		No		Alive	36 months
Kaneko et al. (1999)	10484813	1	Male	35	2805		1	Dead	37 months
		2	Male	37	3308		2	Alive	132 months
		3	Female	35	2922		2	Dead	7 months
		4	Male	34	2558		2	Dead	4 months

Beck et al. (1997)	9364305	1	Male	39	3000	No	3	Alive	8 months
Roggero et al. (1997)	9142307	1	Male	at term	3570	No	2	Dead	29 months
Drumm et al. (1988)	2891946	1	Female	33	1900		1	Dead	11 months
		2	Female	39			1	Dead	22 months
Walker-Smith et al. (1985)	2866310	1	Female					Alive	5 months
Phulware et al. (2019)	31049800	1	Male					Alive	3 months
Cheng et al. (2017)	28899465	1	Female	39	2850		2	Alive	1 month
Ozge et al. (2019)		1	Male	36			2	Alive	6 months
Cegla et al. (1993)	8114773	1							
Rhoads et al. (1991)	1993505	1							
Ukarapol et al. (2001)	11800313	1	Male						
Mao et al. (2016)	27984607	1							
Van Hoeve et al. (2016)	27477384	1	Male						
Paulus et al. (2015)	26057766	1	Male	at term					
Thoeni et al. (2013)	24138727	1							
Poley et al. (2006)	17784640	1							
Morroni et al. (2006)	16609911	1							
		2							
Youssef et al. (2004)	15785408	1							
Lopez et al. (2001)	11339115	1	Male						
Acar et al. (1999)	10770118	1	Female						
Gambarara et al. (1997)	9142308	1	Male						
		2	Male						

		3	Female						
Steininger et al. (1997)	9065586	1							
		2							
Oliva et al. (1994)	8119548	1	Female	at term	2870	No		Alive	53 months
Mendes et al. (2014)	25635218	1	Female	36	2800		3		
Al-Daraji et al. (2010)	21070163	1	Male					Alive	3 months
		2	Famale					Alive	2 months
		3	Female						
		4	Female						
		5	Female					Alive	2 months
		6	Female					Dead	11 months
		7	Female					Alive	1 month
		8	Female						
		9	Male						
		10	Male						
		11	Male					Alive	1 month
		12	Male						
		13	Male					Alive	2 months
		14	Female					Alive	5 months
		15	Male					Alive	4 months
		16	Female					Alive	2 months
		17	Male					Alive	1 month

We searched EMBASE and MEDLINE databases using the following search strings: ((microvill* inclusion disease) OR (microvill* atrophy)) AND case report) to collect all published MVID case reports. Totally, 83 valid case reports reporting on 131 MVID patients were retrieved.
